# Gratitude as Mood Mediates the Effects of a 6-Weeks Gratitude Intervention on Mental Well-Being: *Post hoc* Analysis of a Randomized Controlled Trial

**DOI:** 10.3389/fpsyg.2021.799447

**Published:** 2022-01-14

**Authors:** Ernst Bohlmeijer, Jannis Kraiss, Marijke Schotanus-Dijkstra, Peter ten Klooster

**Affiliations:** Department of Psychology, Health and Technology, University of Twente, Enschede, Netherlands

**Keywords:** gratitude, mediation, well-being, intervention, moderation, mood

## Abstract

There is a gap of knowledge about the extent to which gratitude is indeed the working mechanism of change in gratitude interventions aiming to promote mental well-being. This study explores the mediational role of gratitude as mood in the context of a recently conducted randomized controlled trial on the effects of a 6-week gratitude intervention on mental well-being in comparison with a waitlist control group. Gratitude as mood was measured at 2, 4, and 6 weeks. Both simple and multiple mediation models were conducted as well as various sensitivity analyses. Results showed a gradual increase of gratitude as mood during the intervention. The effects of the 6-week gratitude intervention on mental well-being were mediated by increases of gratitude as mood at 4 weeks but not at 2 weeks. These findings suggest a dose-response relationship for gratitude interventions, but more research is warranted.

## Introduction

Gratitude has been conceptualized as both an emotion (McCullough et al., 2001) and a more trait-like or dispositional attitude toward life (McCullough et al., 2002; Wood et al., 2010). State-like gratitude is an emotional response to the experience of receiving a benefit from other persons or life itself. This specific emotional response is more likely to occur when the act of goodness by others is interpreted as valuable, demanding real effort and based on a sincere motivation to do good (Wood et al., 2008). Some people are more inclined toward appreciating the good in life and in others. Defining characteristics of grateful people are a sense of abundance, a tendency to appreciate small pleasures and to note and appreciate positive contributions of other people to one’s life (Watkins et al., 2003). Awareness of the transience of life and scarcity can be important sources for a tendency to be grateful (Wood et al., 2010; Emmons, 2013).

In addition to gratitude as an emotional state and gratitude as an affective trait, gratitude as mood can be considered as an intermediate level (Rosenberg, 1998; McCullough et al., 2004). Affective traits can be defined as “stable predispositions toward certain types of emotional responding” (Rosenberg, 1998, p. 249). Affective states are typically brief psychophysiological changes. Where traits are by definition harder to influence, affective states are highly dependent on specific events or situations and of short duration. Intermediate moods are more under intentional control than traits (Rosenberg, 1998) and of longer duration than emotions. This longer duration allows “them to influence information processing, physiological reactivity, and other psychological phenomena over relatively long arcs of time” (McCullough et al., 2004, p. 296). In the 6-weeks intervention, evaluated in our study, participants in the intervention group received a new gratitude intervention each week (Bohlmeijer et al., 2020). In addition, they received the instruction to meditate for 5 minutes after awakening each day for 6 weeks on their intention to notice and appreciate small pleasures and benefits and good deeds of others. This instruction was given to promote a grateful mood during the day and thereby increasing the likelihood of experiencing grateful emotions during the day (McCullough et al., 2004).

Researchers have studied the adaptive functions of gratitude. For example, experiencing and expressing gratitude have been found to promote high-quality relationships (e.g., Algoe et al., 2010; Grant and Gino, 2010; Lambert and Fincham, 2011; Algoe, 2012; Algoe and Zhaoyang, 2016; O’Connell et al., 2018). Also, as a positive emotion, gratitude will contribute to a more broadened thought-action repertoire (Fredrickson, 2001; Fredrickson and Joiner, 2002) and to positive spirals building durable physical, cognitive, and social resources promoting both the ability to adapt and mental health (Fredrickson, 2001; Fredrickson and Joiner, 2002). Additionally, gratitude interventions may promote the use of adaptive coping-styles such as positive reframing and the ability to process difficult life-events. Watkins et al. (2015) inferred that exercising gratitude may train cognitive biases that can contribute to well-being. Two studies found that the effect of dispositional gratitude on depressive symptoms was mediated through positive reframing and positive emotions (Lambert et al., 2009, 2012). Finally, gratitude interventions have been found to decrease repetitive negative thinking, which also served as mediator for the effect of the intervention on depression and anxiety (Heckendorf et al., 2019). Another study found that meaningful goal pursuit mediated the effect of a gratitude intervention on anxiety (Otto et al., 2016).

The above studies either demonstrate or suggest that gratitude interventions can instigate various processes of change that in turn mediate their impact on mental well-being and mental illness. Other studies have focused on changes in gratitude itself as a mediational factor. For example, Voci et al. (2019) found dispositional gratitude to mediate the relationship between mindfulness and psychological well-being in both meditators and non-meditators. Bryant et al. (2020) found that state gratitude mediated the effects of savoring valuable life lessons for older adults on life-satisfaction, hope, and self-esteem. However, there is a scarcity of research evaluating the role of gratitude mediating the impact of gratitude interventions on mental health. Deng et al. (2018) found that dispositional gratitude mediated the effects of counting blessings on subjective well-being among Chinese prisoners. This intervention, however, had a duration of only 1 week and measures were only taken before and after the intervention limiting the evidence for true mediation.

Stronger evidence for mediation comes from studies assessing processes of change during the intervention. A mediator is a variable that represents a possible mechanism through which an intervention achieves its desired effects (Kraemer et al., 2002). Mediating variables should be ideally measured after randomizing participants and prior to postintervention measurement. This allows for establishing temporal precedence: a demonstration of the assumption that changes in the mediating variable occurs before changes in the outcome (MacKinnon et al., 2013; Kendall et al., 2017). In this study, we present results on the mediational role of gratitude using data from a randomized controlled trial (RCT) evaluating the impact of a 6-weeks gratitude intervention on mental well-being (Bohlmeijer et al., 2020). In this trial, moderate to large effects on mental well-being and gratitude as mood were found at posttest in comparison to an active control group and a waitlist control group. In this paper we present the results of interim measurements at 2 and 4 weeks in addition to the earlier reported pre- and post-measurements and we investigate whether interim scores on gratitude as mood mediate the effects of the intervention on mental well-being at posttest in comparison with the waitlist control condition and controlling for baseline scores of gratitude as mood. Although the active control condition, acts of self-kindness, also had a significant effect on gratitude as mood, the gratitude intervention was significantly (*p* = 0.027) more effective in increasing grateful mood than acts of self-kindness (Bohlmeijer et al., 2020). Therefore, we chose the delayed intervention (wait-list) group for a purer comparison and to optimize contrast in the assumed underlying working mechanism.

The current study aims to contribute to the current knowledge about gratitude interventions in several other ways as well. First, recent meta-analyses of gratitude interventions, such as the gratitude letter and gratitude list, found limited evidence for small average effect on well-being and distress across studies (Wood et al., 2010; Davis et al., 2016; Dickens, 2017). One explanation is that most studies included in these reviews evaluated single interventions of short duration (1 or 2 weeks). Increasing the dosage and variation of gratitude intervention exercises may contribute to larger effects on mental health (Lyubomirsky and Layous, 2013). Recent trials on the effects of gratitude interventions of longer duration seem to underscore this (Heckendorf et al., 2019; Bohlmeijer et al., 2020). Our study may give further evidence for a dose-response mediation effect of gratitude in order to promote mental well-being. If a longer duration of gratitude interventions yields larger effects on well-being, it is likely that the indirect effect of gratitude is stronger at 4 weeks than at 2 weeks.

Secondly, we chose gratitude as mood as measure of gratitude taking into account that this measure showed the largest effect in the trial (Bohlmeijer et al., 2020) and because we propose that it is a particularly relevant interim, or proxy, outcome for the ultimate effects on well-being of gratitude interventions. Few studies have examined gratitude as mood as the assumed mechanism of change.

To summarize, there is a gap of knowledge about the extent to which gratitude is indeed the working mechanism of change in gratitude interventions aiming to promote mental well-being. This study aims to address this gap by studying the mediational role of gratitude as mood in the context of a RCT.

## Materials and Methods

### Participants

The flow of participants is described in the main RCT article (Bohlmeijer et al., 2020). The sample in this trial was recruited *via* advertisements on Facebook, LinkedIn, an online newsletter of a popular psychology magazine and Dutch regional newspapers. For the current *post hoc* analysis we included the participants in the gratitude intervention and waitlist conditions (*N* = 169). The mean age was 48.7 (SD = 9.4), and a predominant part of the sample was female (*n* = 152, 89.9%), highly (university or higher vocational) educated (*n* = 132, 78.1%) and of Dutch nationality (*n* = 162, 95.6%). About half of the sample was living with children (*n* = 81, 47.9%) and had paid employment (*n* = 97, 57.4%). Sample characteristics did not significantly differ between conditions (all *p*’s > 0.099), except for living with children. Participants receiving the gratitude intervention were more often living with children compared to those receiving the waitlist condition, χ^2^(1) = 5.00, *p* = 0.025 (Table 1).

**TABLE 1:** Sample characteristics (*N* = 169).

	Gratitude intervention (*n* = 85)	Waitlist control (*n* = 84)
Age, *M* (SD)	47.7 (9.5)	49.7 (9.4)
Female gender, *n* (%)	77 (90.6)	75 (89.3)
**Education, *n* (%)**		
Low	2 (2.4)	4 (4.8)
Intermediate	17 (20.0)	14 (16.7)
High	66 (78.6)	66 (78.6)
**Marital status, *n* (%)**		
Married	46 (54.1)	46 (54.8)
Divorced or widowed	17 (20.0)	19 (22.6)
Never been married	22 (25.9)	19 (22.6)
Dutch nationality, *n* (%)	81 (95.3)	81 (96.4)
Living alone, *n* (%)	14 (16.5)	21 (25.0)
Living with children, *n* (%)	48 (56.5)	33 (39.3)
Paid employment, *n* (%)	49 (57.6)	48 (57.1)

### Procedure

The RCT from which the current data was used, was approved by the Ethics Committee of the University of Twente (BCE17240) and registered in the Dutch Trial Register (NTR6786).

In September 2017, advertisements were placed *via* the different media to recruit the participants. The recruitment message was: “Can your well-being use a boost? Join this study on the effects of happiness exercises from the University of Twente for free.” Participants needed to be at least 18 years old, have a sufficient Internet connection and a valid email address, and they had to master the Dutch language to complete questionnaires and follow the intervention instructions. Interested participants applied online for the study. Participants who completed the online informed consent procedure were automatically redirected to an online screening questionnaire.

Recruited participants who gave online informed consent received a screening questionnaire. Eligible participants were at least 18 years old and had no severe depressive or anxiety symptoms as indicated by a score <34 on the Center for Epidemiological Studies Depression (CES-D) questionnaire (Radloff, 1977; Bouma et al., 1995; Santor et al., 1995) and a score <15 on the Generalized Anxiety Disorder-7 (GAD-7) questionnaire (Spitzer et al., 2006; Donker et al., 2009). After completing the online baseline survey, participants were randomly allocated (1:1) *via* randomizer.org – stratified by gender, low, medium, and high education level and non-flourishing – to the 6-week gratitude intervention (*n* = 85) or the waitlist control condition (*n* = 84). Non-flourishing was determined with the Mental Health Continuum-Short Form (MHC-SF) using the cut-off scores as proposed by Keyes (2006). Non-flourishing is classified when participants do no possess high levels of at least one aspect of emotional well-being (e.g., positive affect or life-satisfaction) and at least 6 of the 14 aspects of social and psychological well-being (e.g., social contribution, purpose in life, and autonomy).

### Conditions

The 6-weeks gratitude intervention consisted of psycho-education and evidence-based gratitude exercises which were send by email each week (Emmons and Mccullough, 2003; Emmons and Stern, 2013). Throughout the weeks, participants practiced with counting your blessings, taking another perspective, expressing gratitude (e.g., writing a gratitude letter), grateful memories, writing about gratitude in times of misfortune and gratitude as an attitude in life. Also, participants received questions to trigger reflection about cultivating gratitude (e.g., “*What did you feel when practicing the gratitude exercise of this week?*” and “*What did it mean for your daily activities?*”).

Participants in the waitlist control condition were told that they could choose the activity that fits best to their needs to improve their happiness and well-being after monitoring their normal fluctuations in their level of well-being for 6 weeks. Participants in the waitlist control condition were also invited to fill out the gratitude as mood scale at 2 and 4 weeks, in addition to the questionnaires pre- en post-intervention and at 6-weeks follow-up. After the 6-weeks follow-up, the participants in this condition chose their activity (the gratitude intervention, an acts of kindness intervention or a self-kindness intervention) for which they received the instructions during the following 6 weeks.

### Measures

Mental well-being was measured at baseline and posttest with the 14-item MHC-SF. The MHC-SF assesses emotional, social, and psychological well-being, making this a multidimensional measure of well-being (Keyes et al., 2008). On a continuous scale that runs from 0 (*never*) to 5 (*almost always*), higher mean scores indicate higher levels of well-being over de past 4 weeks. The MHC-SF showed good internal consistency at baseline (Cronbach’s α = 0.88) analogous to prior studies, including a validation study in the general Dutch population and a clinical population (Lamers et al., 2011; Franken et al., 2018).

Grateful mood was assessed during the intervention at 2 and 4 weeks after baseline using four questions derived from McCullough et al. (2004). These questions were: “In the past 24 hours, (1) …I felt grateful”; (2) “…I was consciously aware that life is good for me”; (3) “…I appreciated the simple things in life”; (4) “…I felt grateful for what others do and have done for me in my life.” Answer categories ranged from 1 (*totally disagree*) to 7 (*totally agree*). Higher mean scores indicate a higher level of grateful mood. Cronbach’s alpha in the present study was 0.83 at baseline and 2 weeks after baseline, and 0.90 at 4 weeks after baseline, indicating good reliability.

### Statistical Analyses

Analyses were conducted in Mplus version 7.11 (Muthén and Muthén, 2015). Several mediation models were run to determine the role of gratitude as mood as mediator for the intervention effect on mental well-being. Maximum likelihood estimation was used in all models. The independent variable (*X*) was the categorical group variable (0 = waitlist, 1 = gratitude intervention), and the dependent variable (*Y*) was the observed mean score on the continuous mental well-being variable at posttest in all models.

First, two simple mediation models were fit, in which the observed total scores of gratitude 2 weeks after baseline were included as mediator for the first model, and gratitude 4 weeks after baseline for the second model.

Second, a multiple mediation model was conducted using both mediators in one model to examine whether results of the simple mediation models hold if both gratitude assessments are included in the same model. To control for baseline variance in gratitude, mediators were additionally regressed on baseline scores of gratitude in all mediation models. Total, direct, total indirect, and (specific) indirect effects were calculated for all models (Hayes, 2017). To determine statistical significance of the effects, corresponding 95% bias-corrected confidence intervals were computed using bootstrapping with 5000 resamples. If the 95% bootstrapped confidence interval did not contain zero, we assumed that the effect is significant (Preacher and Hayes, 2008). Completers only (listwise deletion) were used for all models, since bootstrapping is not available for imputed data in Mplus.

Third, two sensitivity analyses were conducted. The multiple mediation model does not account for the fact that the two mediators were assessed at different timepoints which are not statistically independent. Therefore, an additional multiple mediation model was run including both mediators, as well as the autocorrelation between the two mediators. This additional analysis was done because it is possible that including the autocorrelation might change other paths in the mediation models, which might affect conclusions drawn about mediation effects. In addition, it is possible that including completers only in the analyses biases the regression paths, since only a specific group of participants might have continued completing the questionnaires. Therefore, we also conducted all mediation models with imputed data to check whether the estimates substantially differ from the models obtained using completers only. Proportion of missing data was as follows: gratitude 2 weeks after baseline (11.8%), gratitude 4 weeks after baseline (33.7%), gratitude at posttest (21.9%), and mental well-being at posttest (21.3%). For this sensitivity analysis, multiple imputation with 10 data sets with missing data generated from Markov chain Monte Carlo simulation was used (Asparouhov and Muthén, 2010). Since multiple imputation does not allow for the calculation of bootstrapped confidence intervals in Mplus, we used *p*-values instead of 95% bias-corrected confidence intervals to determine significance in this model.

## Results

### Simple Mediation Models

**FIGURE 1:**
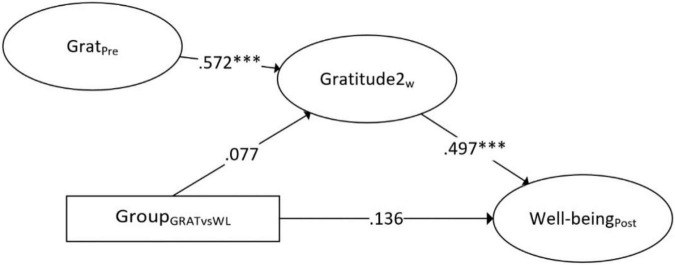
Simple mediation of gratitude 2 weeks after baseline as mediator for the effect of the gratitude intervention versus waitlist on mental well-being (standardized estimates). ^***^p < 0.001.

**FIGURE 2:**
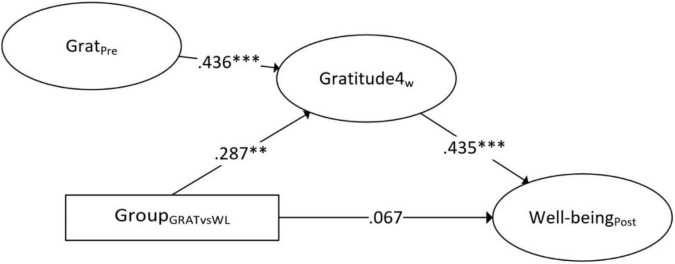
Simple mediation of gratitude 4 weeks after baseline as mediator for the effect of the gratitude intervention versus waitlist on mental well-being (standardized estimates). ^**^p < 0.01, ^***^p < 0.001.

Both simple mediation models are shown in Figures 1, 2. In the simple mediation model with gratitude 2 weeks after baseline as mediator, a significant total effect of group on mental well-being was found (β = 0.18, *b* = 0.26, 95% CI: 0.03–0.47), but no significant direct or indirect effect (both 95% CIs containing zero). This suggests that the gratitude intervention had no significant effect on mental well-being when partialing out the effect of gratitude as mediator, and also that the effect of the gratitude intervention was not mediated through gratitude 2 weeks after baseline. The a-path (i.e., from group to gratitude) was relatively weak and not significant (β = 0.08, *b* = 0.68, 95% CI: −0.52 to 1.82), indicating that gratitude as mood was not impacted after following the intervention for 2 weeks.

The simple mediation model with gratitude 4 weeks after baseline as mediator showed that the total (95% CI: 0.06–0.51) and indirect effect (95% CI: 0.07–0.34) were significant, while the direct effect was not significant (95% CI: −0.16 to 0.34). This suggests that the intervention, similar to the first simple mediation model, had no significant effect on mental well-being while accounting for gratitude as mediator. In contrast to the first simple mediation model, gratitude scores 4 weeks after baseline mediated the effect of the intervention. Compared to the first simple mediation model, a relatively strong significant effect from group to gratitude 4 weeks after baseline was found (β = 0.29, *b* = 3.08, 95% CI: 1.15–4.80), suggesting that the gratitude intervention did have a significant impact on gratitude after following the intervention for 4 weeks, but not after 2 weeks.

### Multiple Mediation Model

**FIGURE 3:**
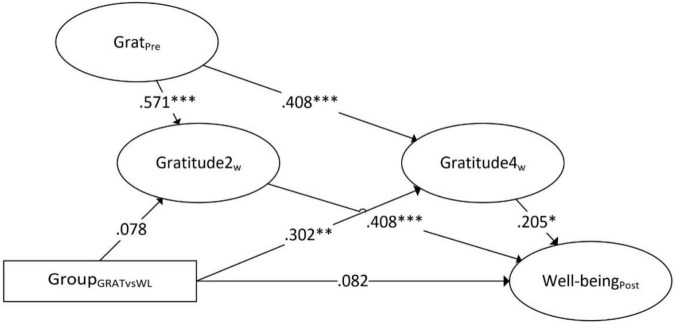
Multiple mediation of gratitude 2 and 4 weeks after baseline as mediators for the effect of the gratitude intervention versus waitlist on mental well-being (standardized estimates). *p < 0.05, ^**^p < 0.01, ^***^p < 0.001.

The multiple mediation model is shown in Figure 3. Including both mediators in the same model revealed relatively similar results compared to the simple mediation models. A significant total effect of group on mental well-being (β = 0.18, *b* = 0.25, 95% CI: 0.04–0.46) and total indirect effect was found (95% CI: 0.02–0.28), while the direct effect was not significant (β = 0.08, *b* = 0.12, 95% CI: −0.11 to 0.33). The specific indirect effects showed that the indirect effect through gratitude 2 weeks after baseline was not significant (95% CI: −0.03 to 0.14), while the indirect effect through gratitude 4 weeks after baseline was significant (95% CI: 0.01–0.23). The estimate of group to gratitude 4 weeks after baseline (*b* = 3.19) fell outside the confidence interval of the effect from group to gratitude 2 weeks after baseline (95% CI: −0.51 to 1.83), indicating that the effect of the intervention on gratitude was significantly stronger after 4 weeks. Outcomes of the simple and multiple mediation models are summarized in Table 2.

**TABLE 2:** Results of simple and multiple mediation models.

	STD. estimate	Estimate	SE	Boot 95% CI	
				Lower	Upper
**Simple mediation: gratitude 2 weeks**					
Paths					
Group → Grat_2w_	0.077	0.684	0.596	–0.515	1.822
Grat_2w_ → well-being	0.497	0.082	0.014	0.056	0.109
Grat_Pre_ → Grat_2w_	0.572	0.525	0.068	0.396	0.663
Total effect	0.175	0.255	0.111	0.033	0.466
Direct effect	0.136	0.199	0.108	–0.014	0.407
Indirect effect	0.038	0.056	0.050	–0.042	0.159
**Simple mediation: gratitude 4 weeks**					
Paths					
Group → Grat_4w_	0.287	3.076	0.932	1.150	4.802
Grat_4w_ → well-being	0.435	0.059	0.012	0.036	0.084
Grat_Pre_ → Grat_4w_	0.436	0.482	0.089	0.320	0.670
Total effect	0.192	0.281	0.115	0.059	0.508
Direct effect	0.067	0.099	0.127	–0.155	0.340
Indirect effect	0.125	0.183	0.066	0.071	0.335
**Multiple mediation: gratitude 2 and 4 weeks**					
Paths					
Group → Grat_2w_	0.078	0.690	0.595	–0.511	1.827
Group → Grat_4w_	0.302	3.187	0.946	1.156	4.925
Grat_2w_ → well-being	0.408	0.066	0.016	0.032	0.097
Grat_4w_ → well-being	0.205	0.028	0.014	0.001	0.055
Grat_Pre_ → Grat_2w_	0.571	0.524	0.068	0.394	0.661
Grat_Pre_ → Grat_4w_	0.408	0.446	0.086	0.281	0.617
Total effect	0.176	0.253	0.110	0.035	0.460
Direct effect	0.082	0.118	0.112	–0.108	0.330
Total indirect effect	0.094	0.135	0.065	0.018	0.277
Specific indirect effects					
Group → Grat_2w_ → well-being	0.032	0.046	0.042	–0.031	0.140
Group → Grat_4w_ → well-being	0.062	0.089	0.053	0.012	0.230

*Boot 95% CI, bias-corrected bootstrapped 95% confidence intervals; Grat_Pre_, gratitude baseline; Grat_2w_, gratitude assessment 2 weeks after baseline; Grat_4w_, gratitude assessment 4 weeks after baseline; SE, standard error; STD, standardized.*

### Sensitivity Analyses

Most estimates were similar when the autocorrelation between the two mediators was included in the multiple mediation model. The total and total indirect effect were still significant, while the direct effect was still not significant. Also, the specific indirect effect through gratitude 4 weeks after baseline remained significant (95% CI: 0.01–0.22), while the specific indirect effect through gratitude 2 weeks after baseline remained non-significant (95% CI: −0.03 to 0.14).

Similar to the models with completers only, using imputed data instead of completers only revealed a non-significant indirect effect in the simple model with gratitude 2 weeks after baseline (*p* = 0.305) and a significant indirect in the model including gratitude 4 weeks after baseline as mediator (*p* = 0.034), but the total effects were not significant anymore in both models (*p* = 0.098 and *p* = 0.083, respectively). The total effect also became non-significant in the multiple mediation model (*p* = 0.106), as well as the specific indirect effect through gratitude 4 weeks after baseline (*p* = 0.097). The total indirect effect also fell just short of significance (*p* = 0.068).

## Discussion

### Main Findings

This is one of the first studies to explore changes in gratitude as mood as a working mechanism in gratitude interventions. We analyzed the results of a 6-week gratitude intervention on gratitude as mood in mental well-being in comparison with a waitlist control condition. The results both from simple and multiple mediation analyses suggest that gratitude as mood after 4 weeks and not after 2 weeks mediated the effect of the gratitude intervention on mental well-being, controlling for baseline gratitude as mood.

Gratitude interventions have shown promise in improving mental well-being (Wood et al., 2010; Davis et al., 2016). Gratitude interventions also have been found to promote adaptive processes such as appreciating small pleasures resulting in experiencing more positive emotions, the use of positive reframing and interpersonal responsiveness and reduce barriers for adaptation such as repetitive negative thinking (Fredrickson, 2004; Lambert et al., 2012; Algoe, 2019; Heckendorf et al., 2019; Bohlmeijer and Westerhof, 2021). However, the question whether changes in gratitude explain the effects of gratitude interventions on well-being has been understudied. The findings from our study suggest that this specific tendency to notice and appreciate the positive in life improved significantly after participating for 4 weeks in a gratitude intervention and that this improvement explains, at least partially, the improvement of mental well-being at posttest.

The findings showed that increases of gratitude as mood mediated the effects on mental well-being at 4 weeks and not at 2 weeks. The effects of the intervention on gratitude as mood were also larger at 4 weeks than at 2 weeks. These findings suggest that a longer duration of a gratitude intervention is needed before the changes in gratitude as mood start to impact the effect of gratitude interventions on mental well-being. Most gratitude interventions that have been studied in RCTs had a duration of 1 or 2 weeks (Davis et al., 2016). Systematic reviews of these studies found evidence for the effectiveness of brief gratitude interventions, but the effect sizes were generally small across studies (Davis et al., 2016). One strategy to increase the impact of positive psychological interventions such as the gratitude intervention is to increase the duration and variety of these interventions (Lyubomirsky and Layous, 2013). Recent trials with gratitude interventions of longer duration and with a variety of interventions indeed found moderate to large effects on measures of gratitude and on mental health (Heckendorf et al., 2019; Bohlmeijer et al., 2020). The findings from the current study suggest a dose-effect relationship for gratitude and well-being. This is in line with a recent comprehensive meta-analysis showing that positive psychological interventions in general of longer duration have larger effects than brief interventions (Carr et al., 2020) and with a systematic review about the effects of psychological interventions in general showing that optimal doses of guided self-help interventions range from 4 to 6 sessions (Robinson et al., 2020). However, more research is needed to confirm a dose-effect relationship of gratitude and well-being. Ideally participants are randomly assigned to gratitude interventions of different duration and the combination of specific gratitude interventions is systematically alternated.

In this study gratitude as mood was used as mediating variable. One advantage is that moods are longer in duration and are more rooted in awareness than emotions. Grateful moods are not about something specific and are assumed to facilitate grateful emotions. Though the precise distinction between moods and emotions is debatable, there is consensus about some distinctive features (Russell, 2003; Beedie et al., 2005). Emotions are considered as acute, specific feelings that arise and dissipate quickly in comparison to moods which are generally lower in intensity and of longer duration. Another important distinction is that emotions are generally triggered by specific events while moods are less contingent on specific events (Rosenberg, 1998; McCullough et al., 2004). Moods can also be distinguished from traits. In comparison to moods, traits are seen as “stable predispositions toward certain types of emotional responding” (Rosenberg, 1998, p. 249). Gratitude as mood is potentially also more controllable and changeable than gratitude as a trait and as emotion. One can remind oneself of one’s intention and motivation to be appreciative of positive aspects of one’s life, to take the various facets of life not for granted and to appreciate beneficial acts of other persons (Emmons, 2013) and thus bring oneself willingly in a grateful mood. In the 6-week intervention that was object of this *post hoc* study, participants were encouraged to start each day with a brief reminder and reflection on their intention to appreciate positive aspects of one’s life. This higher level of controllability may explain why the effects on gratitude as mood were larger in comparison with gratitude as disposition and as emotion (Bohlmeijer et al., 2020). The higher level of controllability makes gratitude as mood a good process measure of gratitude interventions and may explain its mediational role as was found in the current study. Bohlmeijer et al. (2020) also reported that the improvements of gratitude and well-being were maintained at 6 months follow-up suggesting an element of sustainability.

Two sensitivity analyses were conducted in the current study to explore the robustness of the findings. First, we found that including the autocorrelation between gratitude at 2 and 4 weeks after baseline in the multiple mediation model did not have a substantial effect on the conclusions drawn from the mediation analysis. This additional analysis was conducted since it could have been possible that including the autocorrelation decreases the effect group assignment has on gratitude at 4 weeks after baseline. This, in turn, could have weakened the specific indirect effect of gratitude at 4 weeks. Importantly, the results with the autocorrelation included still suggest that the effect of the gratitude intervention on mental well-being is mediated through gratitude at 4 weeks, but not 2 weeks after baseline. Second, when using the conservative technique of multiple imputation for the multiple mediation model, overall conclusions were slightly different compared to the model including complete cases only. As opposed to the models with completers only, the total indirect and specific indirect for gratitude at 4 weeks postintervention both fell short of statistical significance. Nevertheless, the overall tendency found in the models with complete cases remained similar in the models with imputed data. The total indirect effect was relatively strong (compared to the direct effect of group), and the mediation effect was more pronounced for gratitude at 4 weeks postintervention.

### Strengths and Limitations

A strength of the current study is that gratitude as mood as mediating variable was measured twice during the intervention allowing assessment of temporal precedence. To our knowledge this is the first gratitude intervention study to conduct this type of mediational analysis. Also, both simple and multiple models were tested, and various sensitivity analyses were conducted. However, also some important limitations apply. First, higher educated women were over-represented in the current study and the findings cannot be generalized to the general population. Secondly, the percentage of missings was higher at 4 weeks than at 2 weeks. This could indicate a self-selection bias, in the case that only the most motivated participants filled-out the questionnaire at this time-point. In the original RCT article (Bohlmeijer et al., 2020), we found that dropouts were significantly younger. Although our sensitivity analyses revealed that the results from the analyses including all cases did not substantially differ, this might decrease the validity of the findings as the completers seem to differ from people who dropped out. Thirdly, our study included only two interim measurements. To find evidence for mediation more robustly, many more interim measurements of the main variables are warranted. This design could corroborate evidence that changes in gratitude as mood precede changes in well-being.

## Conclusion

The findings of this study show that a 6-week gratitude intervention consistently promotes gratitude as mood over 2, 4, and 6 weeks and that the effects of a 6-week gratitude intervention on mental well-being are mediated by increases of gratitude as mood at 4 weeks and not 2 weeks. These findings suggest that individuals participating in a gratitude intervention should continue practicing gratitude for at least 4 weeks to create an optimal impact on well-being. Some individuals may directly experience the emotional benefits of practicing gratitude and this experience may intrinsically motivate them to sustained practice. Future research could study the reasons why individuals continue or discontinue gratitude interventions of longer duration.

## Data Availability Statement

The original contributions presented in the study are included in the article/supplementary material, further inquiries can be directed to the corresponding author.

## Ethics Statement

The studies involving human participants were reviewed and approved by the Ethics Committee University of Twente (BCE17241). The patients/participants provided their written informed consent to participate in this study.

## Author Contributions

EB and MS-D were responsible for the overall study. JK, PK, and EB were responsible for main analyses. All authors contributed to the manuscript.

## Conflict of Interest

The authors declare that the research was conducted in the absence of any commercial or financial relationships that could be construed as a potential conflict of interest.

## Publisher’s Note

All claims expressed in this article are solely those of the authors and do not necessarily represent those of their affiliated organizations, or those of the publisher, the editors and the reviewers. Any product that may be evaluated in this article, or claim that may be made by its manufacturer, is not guaranteed or endorsed by the publisher.
